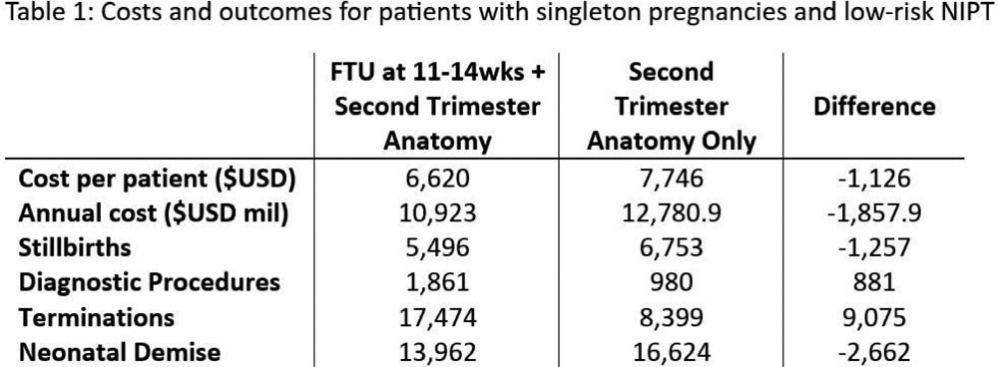# Oral Concurrent Session 6 – Ultrasound and Genetics: Thursday, February 12, 2026 1:00 PM – 3:30 PM

**DOI:** 10.1002/pmf2.70157

**Published:** 2026-02-09

**1:00 PM – 1:15 PM**

## 64 | Rethinking CF Carrier Screening: Comprehensive CFTR Testing vs. Limited Variant Sets

Mona M. Makhamreh^1^; Erica Soster^2^; Jennifer Teicher^2^; April D. Adams^1^; Brandy Firman^3^; Virali Patel^4^; Huda B. Al‐Kouatly^5^

^1^Baylor College of Medicine, Houston, TX; ^2^Labcorp, Westborough, MA; ^3^Thomas Jefferson University, Philadelphia, TX; ^4^Thomas Jefferson University, Philadelphia, PA; ^5^Division of Maternal‐Fetal Medicine, Department of Obstetrics and Gynecology, Sidney Kimmel Medical College at Thomas Jefferson University, Philadelphia, PA, USA, Philadelphia, PA

**Objective**: *CFTR* full gene sequencing with deletion/duplication analysis is not currently standard of care for cystic fibrosis (CF) prenatal carrier testing. We aim to quantify the extent comprehensive *CFTR* testing identifies pathogenic, or likely pathogenic (P/LP) variants missed by current limited *CFTR* variant sets.

**Study Design**: We conducted a retrospective review at a national commercial laboratory (Labcorp) that underwent full *CFTR* gene sequencing with deletion/duplication analysis for routine prenatal screening, between 3/2023 and 1/2024. Identified P/LP variants were compared to three commonly used CF screening variant sets:  ACMG/ACOG‐23, the 23 variant ACMG/ACOG recommended guideline; a commercial 32‐variant set that includes ACMG/ACOG‐23 plus 9 pan‐ethnic and high frequency variants; and 2023 ACMG‐100, an updated set of 100 variants recommended by ACMG. IRB approval was obtained at collaborating institutions.

**Results**: Among 175,858 individuals tested, 6142 (3.5%) had *CFTR* P/LP variants. Of these, 98.9% were detected by sequencing and 1.1% by deletion/duplication analysis. Of the 6142 carriers identified, 3376 (55.0%), 3532 (57.5%), and 4044 (65.8%) would have been detected by ACMG/ACOG‐23, 32‐variant, and ACMG‐100 sets, respectively. A total of 340 distinct P/LP *CFTR* variants were identified. Detection by variant set would have been as follows: 6.8% for ACMG/ACOG‐23, 9.4% for 32‐variant set, 29.4% for ACMG‐100 (Table 1). Thus, 70.6% of P/LP variants would have been missed by the most inclusive *CFTR* variant set.  Notably, 6 of the 10 most frequent P/LP variants (60%) in this cohort were absent from all three variant sets.

**Conclusion**: *CFTR* full gene sequencing analysis outperforms currently recommended variant set‐based approaches, which would have missed **one‐third** to **one‐half** of carriers. This approach captures a broader and more representative variant spectrum, including high‐frequency P/LP variants missed by existing variant sets. Our findings support the adoption of comprehensive *CFTR* testing as the new standard for carrier screening in the U.S. to improve equity and accuracy across diverse populations.

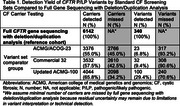

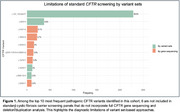

**1:15 PM – 1:30 PM**

## 65 | Development **and Validation of a SNP‐Based cfDNA Test for Vanishing Twin Pregnancies: The VANISH Trial**

Lorraine Dugoff^1^; Sunni L. Mumford^1^; Meredith Rochon^2^; Martin Chavez^3^; Katherine Kohari^4^; Jessica Scholl^5^; Jill Berkin^6^; Ashley S. Roman^7^; Jane Chueh^8^; Samuel Wolf^9^; Kristy Palomares^10^; K. Joseph Hurt^11^; Kelly Gorman^12^; Myra Wick^13^; Zeynep Alpay Savasan^14^; Lawrence D. Platt^15^; Marjorie Treadwell^16^; Abdulla Al‐Khan^17^; Matthew K. Hoffman^18^; Sara Rabin‐Havt^19^; Nicholas Sun^20^; Anastasia Butskova^20^; Wenbo Xu^20^; Vivienne Souter^20^

^1^Perelman School of Medicine at the University of Pennsylvania, Philadelphia, PA; ^2^Lehigh Valley Health Network, Allentown, PA; ^3^NYU Grossman Long Island School of Medicine, Mineola, NY; ^4^Yale School of Medicine and Yale New Haven Hospital, New Haven, CT; ^5^Weill Cornell Medicine, New York, NY; ^6^Icahn School of Medicine at Mount Sinai, New York, NY; ^7^Division of Maternal‐Fetal Medicine, Department of Obstetrics and Gynecology, NYU Grossman School of Medicine, New York, NY; ^8^Stanford University, Stanford University, CA; ^9^Emerald Coast Obstetrics and Gynecology, Panama City, FL; ^10^St. Peter's University Hospital, New Brunswick, NJ; ^11^University of Colorado School of Medicine, Aurora, CO; ^12^University of Kansas School of Medicine, Kansas City, KS; ^13^Mayo Clinic, Rochester, MN; ^14^Corewell Health William Beaumont University Hospital, Royal Oak, MI; ^15^UCLA David Geffen School of Medicine and Cedars‐Sinai Medical Center, Los Angeles, CA; ^16^University of Michigan Hospital, University of Michigan, Ann Arbor, MI; ^17^Hackensack University Medical Center, Hackensack University Medical Center, NJ; ^18^Christiana Care Health System, Newark, DE; ^19^Montefiore Medical Center, Albert Einstein College of Medicine, Bronx, NY; ^20^Natera, Inc., Austin, TX

**Objective**: Develop and validate a SNP‐based non‐invasive prenatal cell free DNA (cfDNA) screening algorithm to identify the cfDNA signal for the living twin in a vanishing twin (VT) pregnancy and assess that twin's aneuploidy risk.

**Study Design**: Prospective study including 108 patients with VTs at 21 US sites (8/21–8/23). Inclusion: >18 years old, >9 wks’ gestation and ultrasound‐confirmed VT. SNP‐based cfDNA screening occurred at enrollment and twice more at 3‐week intervals. Neonatal buccal swabs were collected from the living twin for next generation sequencing, to confirm that the SNPs identified were those of the living fetus. Exclusion: lost to follow‐up, had a pregnancy loss or missing study samples. Using SNP‐based technology, one or two fetoplacental cfDNA signals, in addition to that from the pregnant patient, were identified. Fetal fractions were calculated for each cfDNA signal and an algorithm to identify the living fetus in a VT pregnancy was developed using samples from 47 participants. Concordance between the fetal genotype derived from cfDNA in the maternal plasma and the buccal swab genotype from 61 subjects was used to validate the algorithm in correctly identifying the living fetus. Common aneuploidies and fetal sex were determined for the living fetus in these samples.

**Results**: Median gestational age was 10.8 wks (range: 8.6–16.1) at VT diagnosis. In 57/61 (93.4%; 95% CI: 84.3%, 97.4%) validation cases, we identified the living fetus and made the correct aneuploidy call confirmed by newborn buccal swabs (Figure 1). Aneuploidy results included 56 low‐risk and 1 high‐risk result for trisomy 21. No false positive or false negative results were reported. Validation results were obtained on 55/57 living twins within the first 2 maternal blood draws; 2 cases required 3 draws. At the time the living fetus was identified, 39 samples had two fetal cfDNA signals and 18 had one signal.

**Conclusion**: The SNP‐based algorithm accurately identified the living fetus in VT pregnancies. Aneuploidy risk assessment was validated in all cases where the cfDNA signal for the living twin was identified.

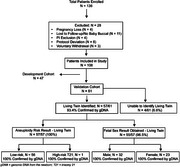

**1:30 PM – 1:45 PM**

## 66 | **AI** Estimation **of Fetal Weight From Ultrasound Sweeps of the Gravid Abdomen**

Teeranan Pokaprakarn^1^; Katelyn J. Rittenhouse^1^; Juan C. Prieto^1^; Margaret Kasaro^2^; Srihari Chari^1^; Yuri Sebastião^1^; Elizabeth M. M. Stringer^1^; Nicole Davis^1^; Mark Klose^1^; Rebecca Ritter^1^; M. Bridget Spelke^1^; Bellington Vwalika^3^; Stephen Cole^1^; Michael R. Kosorok^1^; Jeffrey S.A. Stringer^1^

^1^University of North Carolina at Chapel Hill, Chapel Hill, NC; ^2^UNC Global Projects Zambia, Chapel Hill, NC; ^3^University of Zambia School of Medicine, Chapel Hill, NC

**Objective**: To develop a bedside ultrasound tool that estimates fetal weight from systematically obtained ultrasound sweeps at the time of delivery.

**Study Design**: We trained a deep learning model using data from a prospective AI‐ultrasound cohort and routine clinical imaging from the US and Zambia. Singleton pregnancies ≥22 weeks with standardized 10‐second sweep videos and delivery within two weeks of ultrasound with documented birthweight were included. Data were split at the pregnancy level into training, tuning, and testing sets. We applied further restriction to the held‐out test set, requiring sweeps and biometry to be within one week of delivery.

**Results**: The dataset comprised 7413 pregnancies enrolled between 2018 and 2023, divided into training (*n* = 4744), tuning (*n* = 1220), and testing (*n* = 1449) sets. After applying test set restrictions, 970 visits were analyzed. The AI model achieved a mean absolute error (MAE) of 218g (95% CI: 207, 229 g), outperforming Hadlock IV biometry (MAE 260 g; 95% CI: 248, 273 g; Δ = −42g, 95% CI: −58 g, −27 g). The model estimated fetal weight within 10% of actual birthweight in 71% of cases, compared to 63% for biometry (*p* < 0.001). For detecting macrosomia (birthweight ≥4000 g), the AI achieved an AUC of 95.3% vs. 94.8% for biometry. At 95% specificity, sensitivity was 73% for AI and 62% for biometry. Superior performance also extended to detecting birthweight <2000 g–a common NICU admission threshold.

**Conclusion**: A deep learning model applied to a series of simple ultrasound sweep videos estimated birthweight more accurately than traditional biometry. Performance extended to identifying the clinically significant thresholds of birthweight ≥4000 g and <2000 g. These findings highlight the potential of AI‐enabled point‐of‐care ultrasound to support intrapartum decision‐making and expand access to obstetric diagnostics—particularly in settings where trained sonographers are not available.

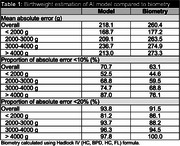

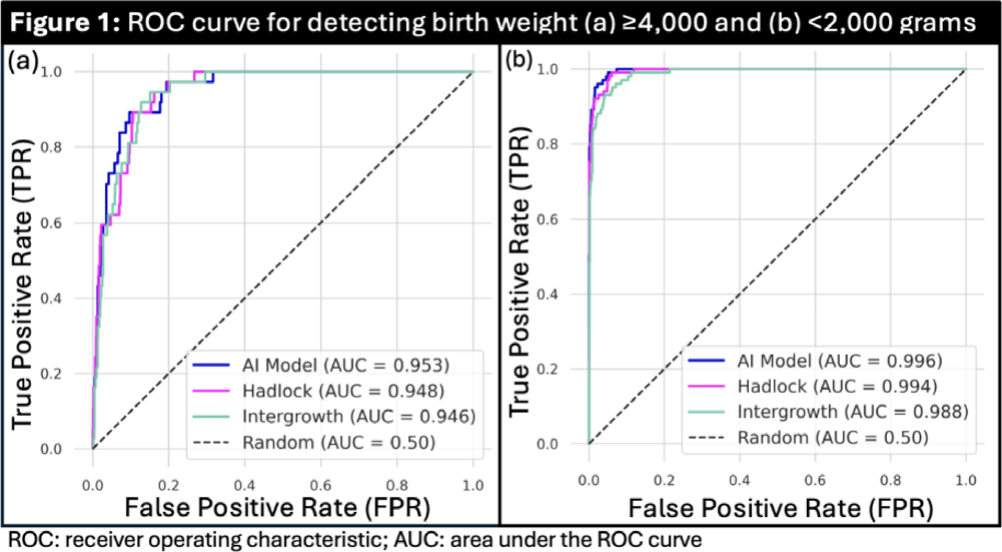

**1:45 PM – 2:00 PM**

## 67 | **Regions of Homozygosity on Prenatal Chromosomal Microarray and Adverse Pregnancy Outcomes**

Victoria Ly^1^; Nicola F. Tavella^2^; Jo Hsuan Lee^3^; Grace M. DiGiovanni^4^; Nina G. Faynshtayn^4^; Barbara Pereira Vera^4^; Shrinkhala Kaphle^5^; Huda B. Al‐Kouatly^6^; Angela T. Bianco^4^; Laura C. Gilroy^7^

^1^Division of Maternal‐Fetal Medicine, Icahn School of Medicine at Mount Sinai, New York City, NY; ^2^Division of Maternal‐Fetal Medicine, Icahn School of Medicine, Icahn School of Medicine at Mount Sinai Hospital, New York, NY; ^3^Icahn School of Medicine at Mount Sinai, Icahn School of Medicine at Mount Sinai, NY; ^4^Icahn School of Medicine at Mount Sinai, New York, NY; ^5^Icahn School of Medicine at Mount Sinai, New York CIty, NY; ^6^Division of Maternal‐Fetal Medicine, Department of Obstetrics and Gynecology, Sidney Kimmel Medical College at Thomas Jefferson University, Philadelphia, PA, USA, Philadelphia, PA; ^7^Mount Sinai Hospital, Mount Sinai Hospital, NY

**Objective**: Prenatal diagnostic chromosomal microarray (CMA) detects regions of allele homozygosity (ROH), suggestive of common parental ancestry. ROH is further delineated as long sequences (>8 Mb) or high density of short sequences. The clinical implications of ROH are unknown, and no guidelines exist for counseling pregnant patients with ROH. This study examined the risk of adverse perinatal outcomes (APO) in pregnancies with ROH on CMA.

**Study Design**: This was a retrospective study of patients with prenatal diagnostic testing at a single tertiary care center from November 2022 to September 2024. CPT codes were abstracted from electronic medical records. We excluded multifetal gestation and congenital anomalies. Individual‐level variables included demographics, medical co‐morbidities, and delivery data. The composite APO included preterm birth, PPROM, FGR, hypertensive disorders of pregnancy, pregnancy loss, and NICU admission. Mann Whitney U‐tests and Student T‐tests compared continuous variables, and χ^2^ and Fisher's exact tests compared categorical variables. Multivariable logistic regression models calculated odds ratios of APO while controlling for individual‐level factors. A simulated randomized trial scenario using Mahalanobis distance matching generated a 1:3 ROH‐to‐no ROH sample.

**Results**: We included 661 patients. 15 (2.3%) pregnancies had ROH on CMA. ROH and no ROH groups differed significantly regarding baseline characteristics (age, racial and ethnic identities, insurance, and gravidity, Table 1). We found an association between ROH and the composite APO (OR = 5.6, 95% CI [1.6–19.6], *p* < 0.01, Table 1). The presence of ROH also increased the odds of FGR (OR = 12.8, 95% CI [3.1–52.8], *p* < 0.001, Table 2). In sub‐analyses, long sequences of ROH also increased the odds for APO (OR = 8.8, 95% CI [2.1–36.9], *p* < 0.01).

**Conclusion**: These results suggest an association between ROH on CMA and adverse pregnancy outcomes. Further investigation with larger, prospective populations of ROH patients is warranted to confirm this association and determine clinical implications.

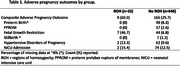

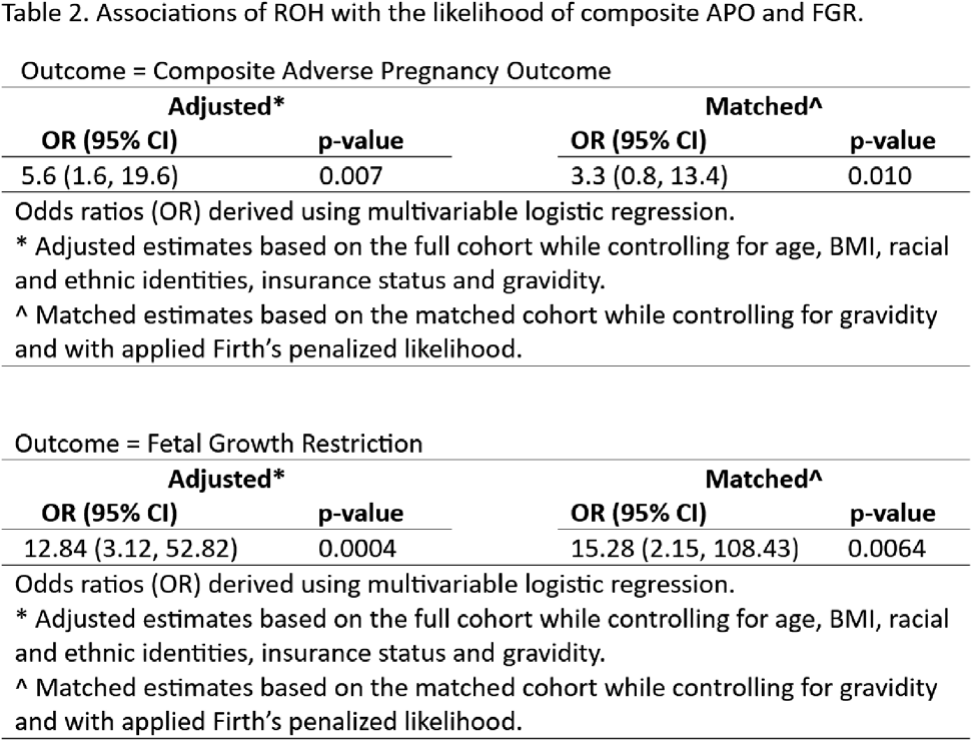

**2:00 PM – 2:15 PM**

## 68 | **Validation of an AI Software for Major CHD Screening in a Prenatal Diagnostic Center**

Jennifer Lam‐Rachlin^1^; Nathan S. Fox^2^; Clementine Roth^3^; Simi Gupta^2^; Shari Gelber^1^; Jessica Spiegelman^2^; Steven R. Inglis^2^; Celia Muoser^2^; Xiangna Tang^2^; Luciana Vieira^2^; Samantha Do^2^; Andrei Rebarber^4^

^1^Icahn School of Medicine at Mount Sinai, Icahn School of Medicine at Mount Sinai, NY; ^2^Icahn School of Medicine, Mount Sinai West, New York, NY; ^3^Carnegie Imaging for Women PLLC, New York, NY; ^4^Icahn School of Medicine, Mount Sinai West, Mount Sinai West, NY

**Objective**: Prenatal diagnosis of severe congenital heart defects (CHDs) is key for improving neonatal outcomes. AI for CHD screening shows strong early evidence and is now entering real‐world validation. In this study, we aim to evaluate the impact of an AI flagging findings suspicious for severe CHD at an AIUM certified Prenatal Diagnostic Center.

**Study Design**: We analyzed anatomy ultrasounds from singleton pregnancies at 18–24 weeks, consecutively acquired over a 7‐month period. Board certified MFM specialists reviewed all scans in person.

An AI software developed by BrightHeart was accessible to sonographers via a cart‐side tablet in real time. The AI first assessed view completeness (defined as ≥2 seconds of 4CH, LVOT and RVOT views in 2D grayscale ultrasound cines). For scans meeting completeness criteria, the AI analyzed all cine clips, and assessed the presence of 8 morphological findings suspicious for severe CHD.

The presence of cardiac abnormalities was established based on clinical reports, including existing follow‐up and pediatric cardiology evaluations.

**Results**: Of 1253 cases, 11 were of non‐severe CHD, 4 of severe CHD (2 transposition of the great arteries; 2 pulmonary stenosis, one with hypoplastic right heart), and 2 with significant extracardiac abnormalities affecting the heart. The AI detected a suspicious finding in all cases of severe CHD and significant extracardiac abnormalities affecting the heart.

Among the 1236 (98.6%) cases without cardiac abnormalities, the AI tool determined all suspicious findings as absent in 1198 cases, yielding a specificity of 96.7% (95% CI: 95.5%–97.6%) and a findings alert rate of 3.3%. The AI specificity was preserved across maternal BMI, fetal presentation, and abdominal scars (see Figure).

**Conclusion**: Real time AI software in clinical practice showed high specificity and reliable CHD detection across diverse factors, showing strong agreement between clinical and AI‐based interpretations. These findings support the clinical usability and generalizability of AI in prenatal cardiac screening and highlight its potential role in improving early CHD detection.

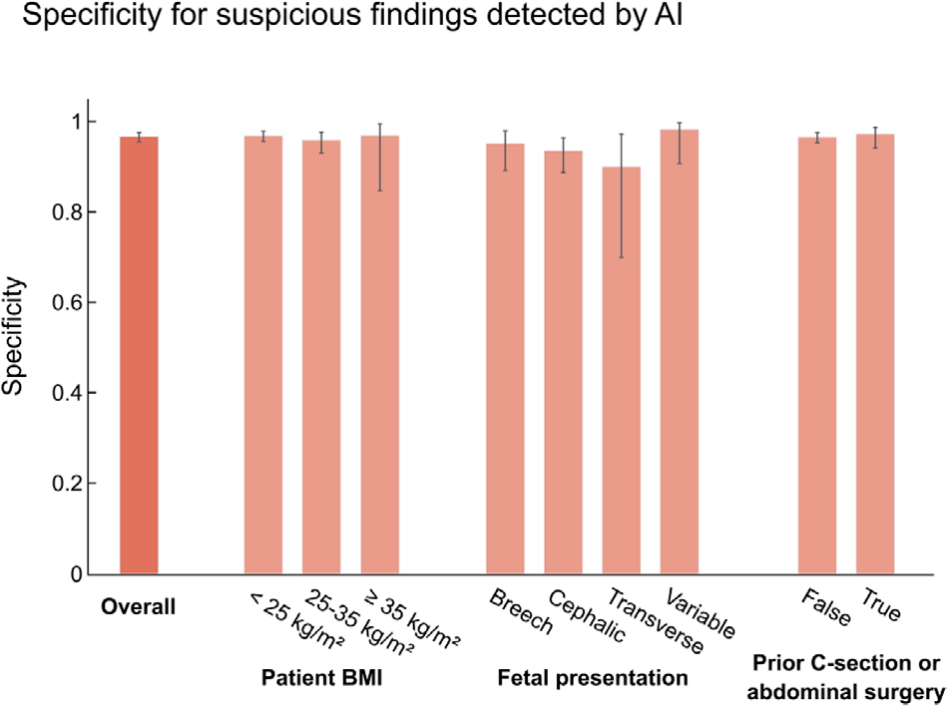

**2:15 PM – 2:30 PM**

## 69 | **Sonographic Quantitative Vascularization of the Anterior Fornix/Cervix for Prediction of Outcomes in Placenta Accreta Spectrum**

Elias Kassir^1^; Edgar A. Hernandez‐Andrade^2^; Donatella Gerulewicz^2^; Sarah T. Mehl^2^; Farah H. Amro^2^; Eleazar E. Soto^1^; Ramesha Papanna^1^; Sean C. Blackwell^2^; Baha M. Sibai^2^

^1^University of Texas Health Science Center in Houston, McGovern Medical School, Houston, TX; ^2^Division of Maternal‐Fetal Medicine, Department of Obstetrics, Gynecology and Reproductive Sciences, McGovern Medical School at UTHealth Houston, Houston, TX

**Objective**: We aim to determine if a novel technique for quantification of vascularity in the anterior fornix/cervix (AFC) on ultrasound (US) images can be used to predict placenta accreta spectrum (PAS) severity and intraoperative blood loss (IOBL) at time of cesarean hysterectomy (CH).

**Study Design**: Prospective cohort of 42 patients with suspected PAS and anterior placenta previa. On transvaginal US done at time of referral, the AFC was defined as a triangle formed by 1) a line following the endocervical canal, 2) a second line from the internal os following the contour of the placenta until its deflexion, 3) a third line from the placental deflexion to the external os (Figure 1). Fixed color Doppler settings were applied: PRF 2.5‐3.4 kHz, low wall motion filter, gain 0.2 dB. Quantitative vascularization (QV) was the percentage of pixels with color Doppler signals within the AFC, estimated with a MatLab^®^ algorithm. Analyses compared QV with placental invasion (ANOVA, post‐hoc tests), and between patients with and without IOBL >1500 mL at time of CH (Student's t‐test). ROC analysis was done for IOBL >1500 mL.

**Results**: Twelve out of 42 patients were found to not have PAS. 30/42 had PAS and underwent CH: 10 had accreta, 11 increta, and 9 percreta. An increase in QV with worsening PAS severity was seen (mean ± SD): no PAS (4.7% ± 3.9%), accreta (12.3% ± 12.9%), increta (18.5% ± 15.9%), and percreta (32.7% ± 19.5%), *p* < 0.001 (Figure 2A). Patients with IOBL >1500 mL (*n* = 9) had higher QV (mean ± SD) (27.5% ± 17.3%) than those with IOBL ≤1500 mL (*n* = 21) (8.7% ± 11.6%), *p* < 0.01 (Figure 2B).

Area‐under‐ROC‐curve to predict IOBL >1500 mL was 0.83 (95% CI 0.69–0.93, *p* < 0.0001). When QV ≥20% the odds ratio for IOBL >1500 mL was 7.91 (95% CI 1.31–47.5; *p* = 0.02); sensitivity 80%, specificity 75%, PPV 57.8%, NPV 89.7%.

All patients without PAS had QV <20%. However, 8/10 patients with accreta, 6/11 with increta, and 2/9 with percreta also had QV <20%.

**Conclusion**: Increased QV in the AFC is associated with greater severity of PAS and increased IOBL at time of CH. In patients with QV ≥20%, consideration may be given to measures to mitigate bleeding at the time of delivery.

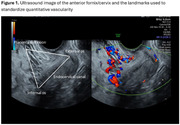

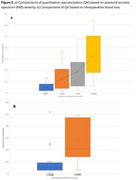

**2:30 PM – 2:45 PM**

## 70 | **Unsupervised Machine Learning of Fetal Myocardial Performance Index Trajectories Identifies FGR and SGA Patterns**

Rebecca Horgan^1^; Erkan Kalafat^2^; Elena Sinkovskaya^1^; Alfred Abuhamad^1^; George R. Saade^1^

^1^Eastern Virginia Medical School at Old Dominion University, Norfolk, VA; ^2^Koc University Hospital, Istanbul, Koc University Hospital, Istanbul, Istanbul

**Objective**: To use unsupervised machine learning approaches on longitudinal trajectories of fetal left ventricular myocardial performance index (MPI) and its association with the risk of subsequent fetal growth restriction (FGR) and small for gestational age (SGA) birthweight.

**Study Design**: This was a prospective cohort study which enrolled patients at ≤13 + 6 weeks’ gestation without fetal, umbilical cord or placental abnormalities. Left ventricular MPI was measured once per trimester using tissue Doppler at 12–13 + 6, 20–21 + 6‐ and 32–33 + 6‐weeks’ gestation. FGR was defined as estimated fetal weight <10th percentile for gestational age. SGA was defined as birthweight <10th percentile for gestational age. Gaussian Mixture Models clustering was applied to identify distinct functional trajectories for left ventricle MPI. Rates of FGR and SGA neonates were compared across clusters using chi‐squared test.

**Results**: Among 306 pregnancies, three distinct MPI trajectories were identified (Figure 1). Cluster 1 consisted of fetuses with low first‐trimester MPI followed by a delayed rise in the third trimester. Cluster 2 included fetuses with persistently elevated MPI across all three time points. Cluster 3 represented fetuses with low first‐trimester MPI that demonstrated early recovery by the second trimester. FGR was more common in Cluster 1 (11.6%) compared to Clusters 2 (2.8%) and 3 (5.4%) (*p* = 0.031). SGA was also most frequent in Cluster 1 (7.2% vs. 0.7% and 0.0%; *p* = 0.001).

**Conclusion**: FGR is characterized by early pregnancy low MPI that does not recover by the second trimester. These findings suggest that fetal cardiac function adapts to an early pregnancy event, likely utero‐placental insufficiency, and that failure of this adaptation is associated with FGR.

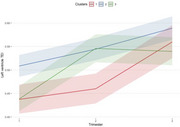

**2:45 PM – 3:00 PM**

## 71 | **pcfDNA Screening With Fetal Fraction Amplification Between 8–10 Weeks: Laboratory Experience With >28k Samples**

Ronit Lebor^1^; D. Claire Miller^2^; Jack Roach^2^; Jhett Bordwell^2^; Seyedmehdi Shojaee^2^; Catherine Wicklund^2^; Summer Pierson^2^; Katie Johansen Taber^2^; Dale Muzzey^2^

^1^Myriad Genetics, Inc., Philadelphia, PA; ^2^Myriad Genetics, Inc., Salt Lake City, UT

**Objective**: In November of 2024, prenatal cell‐free DNA (pcfDNA) screening for chromosomal abnormalities became available beginning at 8 weeks gestation via a whole genome sequencing (WGS) assay that utilizes fetal fraction amplification (FFA). Here, we report on the laboratory experience with pcfDNA screening with FFA between 8w0d and 9w6d gestation for samples received in the first months following availability.

**Study Design**: Data from pcfDNA screens ordered between 11/19/2024 and 07/21/2025 were considered for analysis. Number of orders received, screen‐positive results, and screen failures were calculated. Fetal fraction (FF) medians and first and third quartiles (Q1, Q3) for the 8‐ and 9‐week gestational age period were calculated. Where provided, BMI information was analyzed.

**Results**: During the study period, 28,157 orders were placed for patients between 8w0d and 9w6d. 680 were screen‐positive, for a screen‐positive rate of 2.42%. Screen‐positives included 137 trisomy 21, 43 trisomy 13, 37 trisomy 18, 229 sex chromosome aneuploidies, 275 other autosomal aneuploidies, eight 1p36 microdeletions, three 22q11.2 microdeletions, three 15q11.2 microdeletions, two 4p deletions and four 5p microdeletions. 24 samples were screen‐positive for two or more abnormalities. Results could not be generated for 152 samples, resulting in a screen‐failure rate of 0.54%; 25 samples (0.09%) failed due to insufficient FF. Median FF across the cohort was 15.4% (Q1 = 11.4%; Q3 = 20.2%). For samples ordered in the 8^th^ week of pregnancy, median FF was 13.8% (Q1 = 10.3%; Q3 = 18.2%) and for samples ordered in the 9^th^ week of pregnancy, median FF was 17.1% (Q1 = 12.9%; Q3 = 22.0%). For samples in which BMI could be assessed (*n* = 17,570), the proportion at BMI ≥30 was 35.0% (*n* = 6,237). For those with BMI ≥30, median FF was 12.5% (Q1 = 9.3%; Q3 = 16.1%).

**Conclusion**: Laboratory experience with cfDNA screening for pregnancies between 8–10 weeks gestation demonstrated sufficient FF for analysis while maintaining a low screen‐failure rate across a broad population including patients with high BMI.

**3:00 PM – 3:15 PM**

## 72 | **Incremental Diagnostic Yield of Genome Sequencing for Nonimmune Hydrops Fetalis Spectrum Disorders**

Rozlyn C.T Boutin^1^; Billie R. Lianoglou^1^; Nuriye Sahin‐Hodoglugil^1^; Katie Tick^1^; Carmen M.A. Santoli^2^; Jessica Van Ziffle^1^; Patrick Devine^1^; Ugur Hodoglugil^3^; Pierre Martin^1^; Mary E. Norton^1^; Teresa N. Sparks^1^

^1^University of California, San Francisco, San Francisco, CA; ^2^University of California, San Francisco, University of California, San Francisco, CA; ^3^University of California, San Francisco, San Fraincisco, CA

**Objective**: Exome sequencing (ES) detects a monogenic disorder underlying approximately 1/3 of unexplained nonimmune hydrops fetalis (NIHF) spectrum cases, i.e. those with NIHF or single pathologic fetal fluid collections. Because genome sequencing (GS) can identify additional rare genetic variants not detectable by ES, we sought to determine the incremental diagnostic yield of GS following nondiagnostic ES for NIHF spectrum.

**Study Design**: Retrospective cohort study of pregnancies affected by NIHF spectrum with nondiagnostic karyotype and/or chromosomal microarray (CMA) and ES. Eligible fetal fluid collections included: ascites, pleural or pericardial effusions, skin edema, cystic hygroma, and/or nuchal translucency ≥3.5 mm. Sequencing was performed in a CLIA‐approved laboratory and genetic variants were classified according to the American College of Medical Genetics and Genomics guidelines. Primary outcome was the incremental diagnostic yield of GS for detecting pathogenic or likely pathogenic (P/LP) variants not reported on prior ES and resulting in a new diagnosis. Secondary outcome was the type of variants detected by GS.

**Results**: Overall, 118 fetal samples underwent GS following negative (*n* = 110) or inconclusive (*n* = 8) ES. Cohort characteristics by type of fetal fluid collection are described in Table 1. GS had an incremental diagnostic yield of 2.5% (3/118), identifying four P/LP copy number variants (CNVs, i.e. small deletions or duplications) previously undetected on CMA and ES in three pregnancies that led to a clear genetic diagnosis. Details of the new genetic diagnoses revealed with GS are shown in Table 2. Two new secondary findings and four variants of uncertain significance were also identified with GS in five different pregnancies.

**Conclusion**: GS has a small but clinically relevant diagnostic yield over ES and can identify genetic causes of NIHF spectrum not detectable by ES, particularly CNVs. As interpretation of noncoding regions and complex genomic variations improves, the incremental diagnostic yield of GS over ES is expected to further increase.

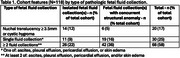

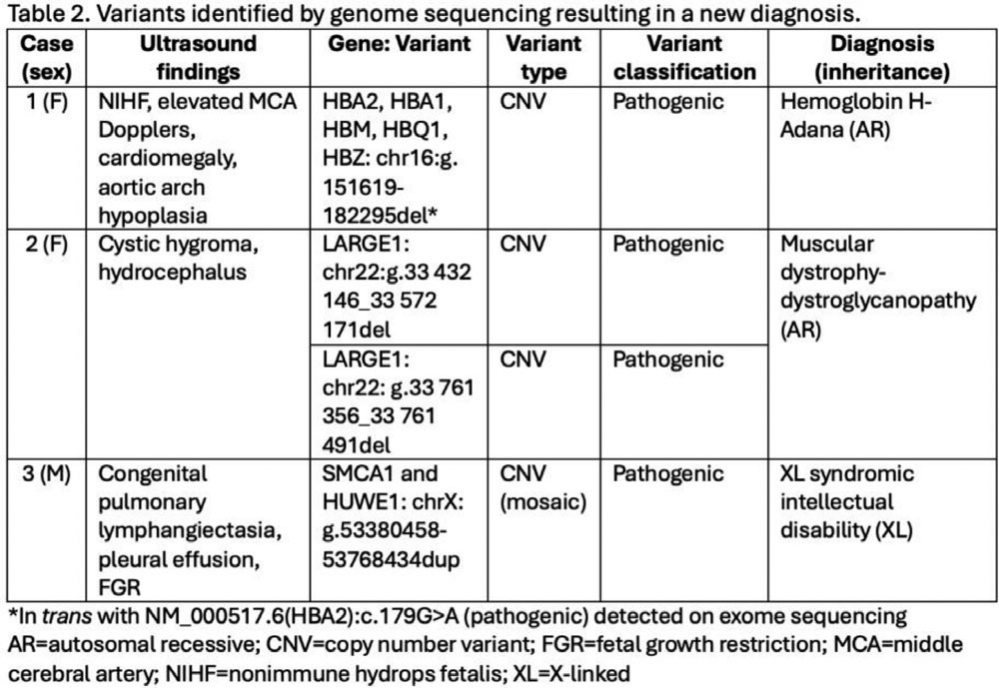

**3:15 PM – 3:30 PM**

## 73 | **Cost‐Effectiveness of First Trimester Ultrasound After Low‐Risk Non‐Invasive Prenatal Testing**

Courtney Hargreaves^1^; Mohak Mhatre^2^

^1^Tufts University School of Medicine, Tufts University School of Medicine, MA; ^2^Tufts University School of Medicine, Boston, MA

**Objective**: Standard first trimester ultrasound (SFTU) at 11‐14wks can detect a number of major or lethal anomalies. However, fewer patients are utilizing SFTU after receiving low‐risk non‐invasive prenatal testing (NIPT) results. This study's aim is to evaluate the cost‐effectiveness of universal SFTU in the context of a low‐risk NIPT result.

**Results**: In our theoretical model, performing an SFTU after low‐risk NIPT was the dominant strategy, resulting in cost savings of $1.86 billion and an increase of 49,500 QALYs, for an ICER of −$35,835/QALY. Performing an SFTU resulted in 881 more diagnostic procedures; 9075 more terminations; and 2662 fewer neonatal demises. Performing an SFTU remains a cost‐effective strategy when there is no access to 2nd trimester abortion care. It is not cost‐effective when there is no access to any abortion care.

**Study Design**: A decision‐analytic model was created using TreeAge Pro to compare outcomes and cost‐effectiveness for two strategies for singleton pregnancies with a low‐risk NIPT result: (1) SFTU at 11‐14 weeks followed by second trimester anatomic survey and (2) second trimester anatomic survey alone. A theoretical cohort of 1.65 million births was used based on the number of annual singleton births in the U.S. with a low‐risk NIPT result. Major or lethal anomalies that could be identified on STFU were included. Outcomes included: prenatal diagnostic testing, procedure‐related loss, spontaneous pregnancy loss, pregnancy termination, and stillbirth. Probabilities, costs, and utilities were derived from the literature. Cost‐effectiveness threshold was set to $100,000/QALY. A societal perspective was assumed. Sensitivity analysis was performed to test the robustness of the model.

**Conclusion**: Following low‐risk NIPT results, first trimester ultrasound to screen for major or lethal fetal anomalies is a cost‐saving approach and results in decreased healthcare costs and improved quality of life. These findings support the importance of continuing to offer first trimester ultrasound despite reassuring NIPT results.